# Municipality-Level Variation in Severe Maternal Morbidity and Association With Municipal Expenditures in New Jersey

**DOI:** 10.1001/jamanetworkopen.2021.35161

**Published:** 2021-11-18

**Authors:** Felix M. Muchomba, Julien Teitler, Lakota Kruse, Nancy E. Reichman

**Affiliations:** 1 Rutgers, The State University of New Jersey, New Brunswick; 2 Columbia University, New York, New York; 3 New Jersey Department of Health, Trenton

## Abstract

**Importance:**

Severe maternal morbidity (SMM) is a major risk factor for maternal mortality, yet little is known about geographic variation in SMM or factors associated with geographical variation at the local level. Municipal governments incur substantial expenditures providing services that are an essential part of residents’ lives, but associations between municipal expenditures and SMM have not been previously examined.

**Objective:**

To investigate variation in rates of SMM across municipalities in New Jersey, the contributions of individual-level characteristics and municipal expenditures to that variation, and associations between municipal expenditures and SMM.

**Design, Setting, and Participants:**

This cross-sectional study analyzed 2008 to 2018 New Jersey birth files linked to maternal hospital discharge records and US Census municipal expenditures data. The birth files contain all birth records for New Jersey, and hospital discharge records contain information from all in-patient hospitalizations in New Jersey over the study period. Birth records were matched to maternal discharge records and expenditures data. Data were analyzed from August 2020 to August 2021.

**Exposures:**

Individual-level characteristics and per capita municipal expenditures on education; public health; fire and ambulance; parks, recreation, and natural resources; housing and community development; public welfare; police; transportation; and libraries.

**Main Outcomes and Measures:**

SMM was identified using diagnosis and procedure codes developed by the Centers for Disease Control and Prevention to measure SMM.

**Results:**

Of 1 001 410 individuals (mean [SD] age, 29.8 [5.9] years; 108 665 Asian individuals [10.9%]; 147 910 Black individuals [14.8%]; 280 697 Hispanic individuals [28.0%]; 447 442 White individuals [44.7%]) who gave birth in New Jersey hospitals from 2008 to 2018, 19 962 individuals (2.0%) had SMM. There was substantial municipality-level variation in SMM that was not fully explained by demographic characteristics. Municipal expenditures on fire and ambulance, transportation, health, housing, and libraries were negatively associated with SMM; $1000 higher annual expenditures per capita in these categories were associated with 35.4% to 67.3% lower odds of SMM (odds ratios, 0.33 [95% CI, 0.15-0.72] to 0.65 [95% CI, 0.46-0.91]). Expenditures on police were positively associated with SMM (odds ratio, 1.15 [95% CI, 1.04-1.28]).

**Conclusions and Relevance:**

The findings in this study regarding associations between spending on various types of services at the municipal level and SMM, holding constant overall spending, population size, and socioeconomic status at the municipal level, indicate that municipal budget allocation decisions were associated with SMM rates and point to the importance of future research investigating potential causal connections.

## Key Points

**Question:**

Are expenditures at the municipal level associated with severe maternal morbidity (SMM), a major determinant of maternal mortality?

**Findings:**

This cross-sectional study including 1 001 410 births from 2008 to 2018 used birth files linked to maternal hospital discharge files from New Jersey and found that municipal expenditures on fire and ambulance, transportation, health, housing, and libraries were negatively associated with SMM, whereas expenditures on police were positively associated with SMM.

**Meaning:**

These findings suggest that budget allocation decisions made by municipalities may have consequences for SMM rates and potentially maternal mortality.

## Teaser

This cross-sectional study examines the associations of variations in municipal-level expenditures with rates of severe maternal morbidity in New Jersey.

## Introduction

The United States has one of highest rates of maternal mortality among high-income countries.^1^ Severe maternal morbidity (SMM), defined by the Centers for Disease Control and Prevention (CDC) as unintended outcomes of labor and delivery that result in significant short- or long-term consequences to a woman’s health,^2^ is a major determinant of maternal mortality.^3^ In 2015, 146.6 of every 10 000 women hospitalized for a delivery experienced SMM.^4^ Besides being an important women’s health outcome, SMM can lead to disruptions in mother-infant bonding, which can compromise children’s social and emotional development.^5^ SMM also confers substantial economic costs to families, employers, insurers, and communities.^6^

Rates of SMM are generally reported at the national, census regional, or state level,^2,4^ with few available robust prevalence estimates at more local levels. Identification of geographic patterns in SMM at finer levels of geography is needed for pinpointing problem areas, identifying structural determinants, identifying potential area-level buffering and exacerbating factors, and targeting of interventions in devolved health systems. One study of New York, New York, neighborhoods found place-based disparities in SMM and highlighted priority communities for intervention efforts.^6^ To our knowledge, there have been no previous studies of within-state geographic variation in SMM.

Past research has found associations between SMM and area-level characteristics, such as poverty rate, median income, high school completion rate, and urbanicity, as well as hospital of delivery,^7,8^ suggesting that SMM could potentially be influenced by area-level expenditures on services that are directly or indirectly related to health. However, while expenditures by state and county governments have been associated with infant health (low birth weight and infant mortality)^9^ and other population health outcomes,^10,11^ associations between government expenditures at any level and SMM have rarely been investigated. An exception is a study that found lower risk for SMM in states that covered medically necessary abortion in their Medicaid programs.^12^

Municipal governments in the US spend more than $600 billion per year on services (including education, transportation, public health, recreation, and police), which is 46% higher than total county-level spending in the same categories.^13^ Because the services provided by municipalities tend to be part of residents’ daily lives, municipal governments are arguably closer to the people.^14^ Consequently, to the extent that government expenditures are associated with SMM, the associations may be more readily observed at the municipality level than at the county or state level. As far as we know, associations between municipal expenditures and health outcomes in the US have not previously been explored.

In this study, we documented the variation in SMM rates across municipalities in the state of New Jersey, evaluated the associations of individual-level characteristics and municipal expenditures with that variation, and estimated associations between specific types of municipal expenditures and SMM. We focused on 9 categories of expenditures that have been associated with population health outcomes and are plausibly associated with maternal health based on past literature or theory: education; public health; fire and ambulance; parks, recreation, and natural resources; housing and community development; public welfare; police; transportation; and libraries, hypothesizing that all categories except police would be negatively associated with SMM.

## Methods

This study was approved by the institutional review boards of Rutgers University and Rowan University (the institutional review board of record for the New Jersey Department of Health). Both institutions approved a waiver of informed consent under the Common Rule (45 CFR 46.116_Section [f]). This report followed the Strengthening the Reporting of Observational Studies in Epidemiology (STROBE) reporting guideline for cross-sectional studies.

### Data Sources

Our analysis is based on individual-level New Jersey birth records on all live births in the state from 2008 to 2018, linked to mothers’ hospital discharge records and area-level data. Following previous research on SMM, we linked the birth records to hospital discharge records from any maternal hospitalization up to 42 days^15,16^ postpartum.^17,18,19^ We kept a single birth record for each delivery regardless of plurality and limited our analysis to births in New Jersey to mothers who resided in New Jersey. We used probabilistic matching to link the records to hospital discharge files using the mother’s name, date of birth, residential address, and hospitalization dates. We successfully matched 94% of birth records to at least 1 maternal discharge record. The birth records were geocoded, allowing us to link each record with municipality- and county-level data from the 2002 and 2007 Censuses of Governments and pooled 2005 to 2009 American Community Surveys.

### Outcome

The outcome of interest was whether the mother experienced SMM. The hospital discharge records included diagnosis and procedure codes using the *International Classification of Diseases, Ninth Revision, Clinical Modification* (*ICD-9-CM*) for years prior to 2016, or the *International Statistical Classification of Diseases, Tenth Revision, Clinical Modification *(*ICD-10-CM*) for 2016 and later. We used the *ICD-9-CM* (in 2008-2015 records) and *ICD-10-CM* (in 2016-2018 records) codes to identify mothers with SMM based on the CDC criteria, which include 16 codes for possible life-threatening diagnoses and 5 codes for life-saving procedures (eTable 1 in the Supplement).^2^

### Birth- and Municipal-Level Characteristics

Birth-level factors, obtained from the birth records, were maternal age, self-reported race and ethnicity, educational attainment, parity, and year of delivery (categories for each year between 2008-2018). Race and ethnicity were included in our analyses to account for the cumulative impact of entrenched racism in the US. The municipality-level factors were expenditures per capita and population size obtained from the Censuses of Governments, and deprivation (eAppendix 1 in the Supplement). The Census of Governments is conducted every 5 years and includes information for all US counties and municipalities. We used the 2002 and 2007 Censuses of Governments, which were collected between 1 and 6 years prior to the period of our study, to establish temporality. Alternative specifications used 2007, 2012, and 2017 Censuses of Governments. We converted the levels of annual government expenditures overall and in 9 specific categories to per capita amounts and inflated them to 2019 dollars (eTable 2 in the Supplement). We calculated the mean of 2002 and 2007 expenditures for each locality and expenditure category to minimize the influence of unusually large, 1-time expenditures. Expenditures were divided by 1000 in the regression analysis to ease interpretation.

### Statistical Analysis

First, we created choropleth maps to visualize the distribution of SMM rates (SMM events per number of births) across all 562 municipalities during the study period (eAppendix 2 in the Supplement). We also generated a choropleth map of SMM rates adjusted for birth-level factors to visually assess their contribution to municipal-level variation in SMM rates.

Next, we estimated multilevel logistic regression models to investigate the extent to which individual- and basic municipality-level factors explain municipality-level variation in SMM. We fit 5 models with 2 random intercepts, mother and municipality, to adjust for clustering at these levels. First, we fit a random intercepts–only model to obtain the municipality-level variation (ie, the variance estimate of the municipality random effect along with the median odds ratio). We then included birth-level or municipality-level factors to estimate the associations between factors at those levels and SMM and estimate the proportional change in variance (ie, proportion of municipality-level variation that could be explained by factors at each of the 2 levels). The last model included both birth- and municipal-level factors. A complete case analysis was used because the level of missing data was low, with the highest proportion (0.6%) being for education.

Finally, we fit similar multilevel logistic regression models to estimate the associations between SMM and municipal expenditures per capita in 9 categories. Separate models were estimated for each of the expenditure categories, controlling for birth-level factors and municipal total expenditures per capita, population size, and deprivation. Each model also controlled for county-level expenditures per capita in the same expenditure category to account for correlations or tradeoffs between municipal and county budgets. There were no municipal-level expenditures on hospitals and hospital care in New Jersey in 2007. Therefore, we performed sensitivity analysis in which we additionally controlled for county expenditures on hospitals and hospital care in all models. We also conducted a post hoc analysis in which spending on categories that were too small across most municipalities to be studied with statistical precision were recoded into binary variables representing whether the municipality had greater than the median expenditure.

The analysis and map drawing were conducted using Stata statistical software version 17 (StataCorp). Details of our test for spatial autocorrelation are in eAppendix 3 in the Supplement. Statistical significance was set at *P* < .05, and all tests were 2-sided. Data were analyzed from August 2020 to August 2021.

## Results

Our sample included 1 001 410 births to women of mean (SD) age 29.8 (5.9) years, residing in 562 municipalities, with 108 665 non-Hispanic Asian individuals (10.9%), 147 910 non-Hispanic Black individuals (14.8%), 280 697 Hispanic individuals (28.0%), and 447 442 non-Hispanic White individuals (44.7%). The rate of SMM in New Jersey during 2008 to 2018 was 199.3 per 10 000 births (Table 1). Mean (SD) total municipal expenditure was $2159 ($1581) per capita. The largest expenditure categories of those we considered were education (mean [SD], $353 [$1010] per capita) and police (mean [SD], $337 [$222] per capita), which together represented approximately one-third of total municipal expenditures. In contrast, municipalities spent a mean (SD) of $7 ($16) per capita on public welfare, less than 0.5% of total spending.

**Table 1.** Birth-Level and Municipality-Level Characteristics of the Sample^a^

Characteristic	No. (%) (N = 1 001 410)
**Birth level**	
Severe maternal morbidity conditions, No.	
Any	19 962 (2.0)
1	17 064 (1.7)
2	1886 (0.2)
≥3	1012 (0.1)
Maternal age, y	
Mean (SD)	29.8 (5.9)
<20	45 602 (4.6)
20-34	737 312 (73.6)
≥35	218 496 (21.8)
Parity	
1	397 687 (39.7)
2	338 536 (33.8)
≥3	265 187 (26.5)
Race and ethnicity	
Hispanic	280 697 (28.0)
Non-Hispanic	
Asian	108 665 (10.9)
Black	147 910 (14.8)
White	447 442 (44.7)
Other or mixed^b^	16 696 (1.7)
Maternal education attainment at birth	
<High school	119 708 (12.0)
High school	263 467 (26.3)
Some college	203 184 (20.3)
≥College	415 051 (41.4)
**Municipality level**	
Expenditures per capita, mean (SD), $^c^	
Total	2159 (1581)
Education	353 (1010)
Libraries	36 (36)
Public welfare	7 (16)
Public health	31 (37)
Fire and ambulance	121 (134)
Police	337 (222)
Housing and community development	145 (209)
Parks and recreation	54 (60)
Transportation	106 (68)
Deprivation index, mean (SD)	0.84 (1.51)
Population size, mean (SD)	61 045 (68 928)

^a^ New Jersey birth records and New Jersey hospital discharge records for 2008 to 2018 were merged with municipal government expenditures obtained from the 2002 and 2007 Censuses of Governments of the US Census Bureau. Sample excludes 47 255 New Jersey residents who delivered outside the state.

^b^ Other race and ethnicity included individuals who identified as American Indian or Alaska Native, Native Hawaiian, Guamanian or Chamorro, Samoan, or other Pacific Islander, or who specified any other racial group that was not Asian, Black, or White.

^c^ Annual amounts in constant 2019 US dollars.

**Figure 1.**
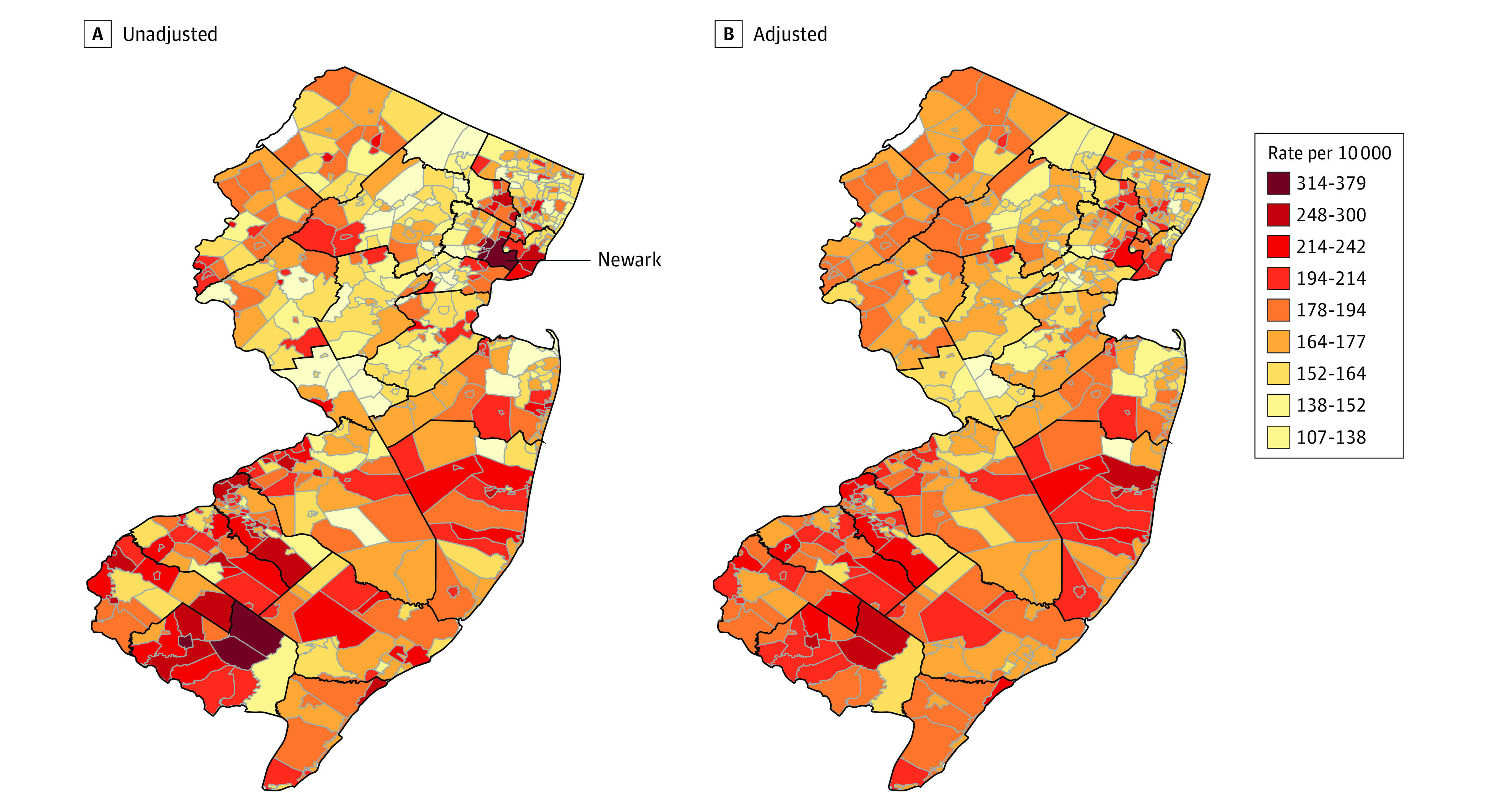
Municipality-Level Severe Maternal Morbidity Rates in New Jersey, 2008-2018 A, Empirical Bayes smoothed estimates; B, Empirical Bayes smoothed estimates adjusted for maternal age, race and ethnicity, educational attainment, and parity. Black lines are county boundaries. Gray lines are municipality boundaries.

There was substantial variation in SMM rates across municipalities (Figure 1A). The rate ranged from 107.5 (95% CI, 80.0-144.6) per 10 000 births in Westfield, an affluent suburban municipality, to 378.5 (95% CI, 330.0-434.0) per 10 000 births in Bridgeton, a low-income city in the southern part of the state. Northern municipalities had relatively low SMM rates and southern municipalities had relatively high rates, with some exceptions (eg, Newark, which is highlighted in Figure 1A, is a city in northern New Jersey with a high SMM rate). Substantial within-county spatial variation would have been masked had we focused on county-level rates instead of municipal-level rates. Finally, adjusting for parity, race and ethnicity, age, and educational attainment at the time of delivery left the overall pattern mostly unchanged, although the rates in outlier municipalities were pulled closer to the middle range, indicating that these characteristics explain little of the municipal variation in SMM (Figure 1B).

In model 1, the median odds of SMM were 31% higher if a mother resided in a municipality with a higher vs lower prevalence of SMM (median OR, 1.31 [95% CI, 1.27-1.35]) (Table 2). In model 2, racial and ethnic composition explained 45.1% of the municipality-level variation. Further adjustment in model 3 by maternal age, education, and parity increased the proportion of variation explained to 49.0%. In model 4, municipality-level covariates explained 49.4% of the variation. Individual- and municipality-level covariates jointly explained 57.8% of the variation in model 5, indicating independent contributions of individual- and municipality-level factors to municipality-level variation in SMM rates.

**Table 2.** Multilevel Models Examining Associations Between Severe Maternal Morbidity and Individual- and Municipality-Level Characteristics in New Jersey From 2008 to 2018^a^

Variable	OR (95% CI)				
	Model 1	Model 2	Model 3	Model 4	Model 5
**Fixed parameters**					
Race/ethnicity					
Hispanic	NA	1.33 (1.27-1.39)	1.23 (1.17-1.29)	NA	1.20 (1.14-1.25)
Non-Hispanic					
Asian	NA	1.07 (1.00-1.13)	1.13 (1.06-1.20)	NA	1.12 (1.05-1.19)
Black	NA	2.12 (2.02-2.23)	2.01 (1.92-2.12)	NA	1.95 (1.86-2.06)
White	NA	1 [Reference]	1 [Reference]	NA	1 [Reference]
Other or mixed^b^	NA	1.38 (1.23-1.55)	1.33 (1.18-1.49)	NA	1.31 (1.16-1.47)
Maternal age, y					
<20	NA	NA	0.98 (0.91-1.05)	NA	0.98 (0.91-1.05)
20-34	NA	NA	1 [Reference]	NA	1 [Reference]
≥35	NA	NA	1.38 (1.27-1.49)	NA	1.39 (1.28-1.50)
Education					
<High school	NA	NA	1 [Reference]	NA	1 [Reference]
High school	NA	NA	0.91 (0.87-0.96)	NA	0.92 (0.87-0.96)
Some college	NA	NA	0.81 (0.77-0.86)	NA	0.82 (0.78-0.87)
≥College	NA	NA	0.73 (0.69-0.78)	NA	0.75 (0.71-0.80)
Parity					
1	NA	NA	1 [Reference]	NA	1 [Reference]
2	NA	NA	0.73 (0.70-0.76)	NA	0.73 (0.70-0.76)
≥3	NA	NA	1.12 (1.08-1.17)	NA	1.12 (1.08-1.17)
Municipality-level variables					
Deprivation	NA	NA	NA	1.20 (1.16-1.23)	1.09 (1.06-1.12)
Total expenditure per capita^c^	NA	NA	NA	0.98 (0.96-1.00)	0.98 (0.96-1.00)
Population size	NA	NA	NA	1.00 (1.00-1.00)	1.00 (1.00-1.00)
**Random parameters**					
Mother-level variance	2.01 (1.83-2.21)	1.95 (1.77-2.14)	1.95 (1.77-2.15)	2.01 (1.83-2.21)	1.95 (1.77-2.15)
Municipality-level variance	0.08 (0.06-0.10)	0.04 (0.03-0.06)	0.04 (0.03-0.05)	0.04 (0.03-0.05)	0.03 (0.03-0.05)
Proportional change in variance, %^d^	0 [Reference]	45.1	49.0	49.4	57.8
Municipality-level median OR (95% CI)^e^	1.31 (1.27-1.35)	1.22 (1.19-1.26)	1.21 (1.18-1.25)	1.21 (1.18-1.24)	1.19 (1.16-1.23)

Abbreviations: NA, not applicable; OR, odds ratio.

^a^ Model 1 was a random intercepts–only model; model 2 additionally adjusted for race and ethnicity; model 3 additionally adjusted for other birth-level factors; model 4 was model 1 adjusted for municipality-level factors; and model 5 adjusted for both birth- and municipal-level factors. Models also control for year of delivery (indicators for each year between 2008-2018).

^b^ Other race and ethnicity included individuals who identified as American Indian or Alaska Native, Native Hawaiian, Guamanian or Chamorro, Samoan, or other Pacific Islander, or who specified any other racial group that was not Asian, Black, or White.

^c^ Annual amounts in constant 2019 thousands of dollars.

^d^ Proportional change in variance is calculated by subtracting the adjusted (models 2-5) municipality-level variance from the baseline (model 1) municipality-level variance and dividing by the baseline municipality-level variance.

^e^ 95% CIs for median ORs calculated assuming asymptotic normality.

Compared with White women, Black women had 2-fold higher odds of SMM (OR, 2.12 [95% CI, 2.02-2.23]), while Hispanic women had 33% greater odds (OR, 1.33 [95% CI, 1.27-1.39]), and Asian women had 7% greater odds (OR, 1.07 [95% CI, 1.00-1.13]). Adjusting for maternal age, education, parity (model 3), and municipality-level factors (model 5) reduced the gap between White and Black women by between 5% and 8%, indicating that racial disparities in SMM only partly reflected differences in other birth-level characteristics and municipality deprivation. The corresponding gap between White and Hispanic women was also reduced, while that with Asian women increased (Table 2). Higher educational attainment was associated with reduced odds of SMM (eg, college or higher education vs less than a high school education: OR, 0.75 [95% CI, 0.71-0.80]; *P* for trend < .001). Of the municipal-level covariates, deprivation was associated with SMM: a 1-SD increase in deprivation was associated with 9% to 20% greater odds of SMM.

**Figure 2.**
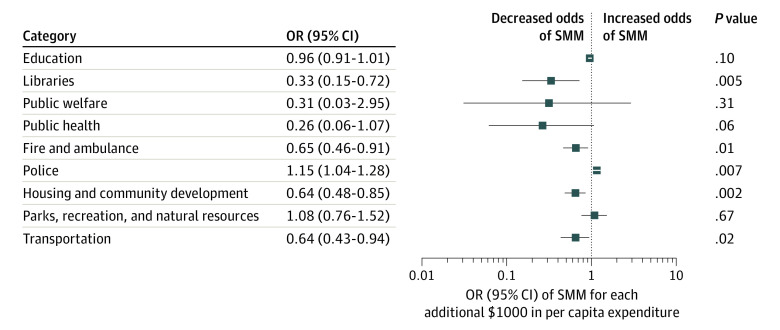
Associations of Severe Maternal Morbidity of Each Additional $1000 Per Capita Spending by Municipality of Residence in New Jersey From 2008 to 2018 Model estimates are presented in eTable 3 in the Supplement.

Municipal expenditures on libraries, fire and ambulance, housing and community development, and transportation were negatively associated with SMM, controlling for age, race and ethnicity, educational attainment, parity, year, total municipal expenditures, municipality deprivation, municipality population size, and county expenditure in the corresponding spending category (Figure 2; eTable 3 in the Supplement). Each additional $1000 annual expenditure per capita in these categories was associated with 35.4% to 67.3% lower odds of SMM (ORs, 0.33 [95% CI, 0.15-0.72] to 0.65 [95% CI, 0.46-0.91]). In contrast, greater municipal spending on police was associated with higher odds of SMM (OR, 1.15 [95% CI, 1.04-1.28]). Point estimates suggested $1000 higher spending per capita on public health was associated with 74.4% lower odds of SMM but the 95% CIs were wide, which could be because most municipalities spent little on public health and thus, we were unable to estimate associations with precision. We further addressed this issue by conducting a post hoc analysis in which municipal spending on public health was recoded into a binary variable representing whether the municipality had greater than the median expenditure (>$16 per capita). The results indicated that odds of SMM among women residing in municipalities with greater than median public health expenditures were 9% lower (OR, 0.91 [95% CI, 0.86-0.97]) compared with municipalities with lower expenditures. Results remained robust to controlling for county-level expenditures on hospitals and hospital care. Model coefficients in which the outcome was SMM excluding blood transfusion were similar to those for blood transfusion (eTables 4-7 in the Supplement). Alternative specifications yielded similar conclusions (eAppendix 4, eTable 8, and eTable 9 in the Supplement).

## Discussion

This cross-sectional study investigated geographical variation in SMM within New Jersey, a state with some of the poorest maternal health indicators in the nation, and associations between various types of municipal expenditures and SMM. The analysis revealed municipality-level variation in SMM that was only partly explained by sociodemographic compositional factors. Mothers’ race and ethnicity, which accounted for 45.1% of the variation in SMM across municipalities, was a particularly strong factor, in line with a cumulative impact of structural racism. Municipal expenditures on fire and ambulance, libraries, housing and community development, and transportation were associated with lower odds of SMM: $1000 per capita higher expenditure per year in these categories was associated with between 35.4% and 67.3% lower odds of SMM, controlling for socioeconomic status, total expenditures, and population size. Additionally, greater than median municipal expenditures on public health were associated with lower odds of SMM. In contrast, municipal expenditures on police were associated with greater odds of SMM. These findings are consistent with previous research documenting protective associations with state and county expenditures on libraries, public health, housing, fire and ambulance, and highways for infant health^9^ and other population health outcomes.^10,11^

Public health spending can provide resources and community health programs that promote healthy pregnancy and reduce chronic conditions that are associated with SMM, such as obesity, hypertension, and diabetes.^20^ Fire and ambulance spending can provide better emergency transportation medical services.^20^ Municipal spending on public transportation can connect individuals to jobs, grocery stores, health care sites, and other resources that may improve health.^20^ Bus shelters, sidewalks, crosswalks, and bike lanes can facilitate physical activity, which may reduce obesity and chronic health conditions.^21^

Public libraries are an important resource for information and the sole access to computers and the internet for many adults with low income.^22^ Libraries also provide opportunities for skills training and social engagement; for example, many public libraries offer English as a second language programs, which could be important for health literacy.^23^

A large body of research has documented health benefits of stable and safe housing^24,25^ and previous studies have found associations between housing instability and poor maternal self-rated health, maternal depression, and low birth weight.^26^ Municipal housing investments supplement inadequacies in federal housing programs and could provide a safe space during pregnancy and recovery from childbirth to store medications, prepare nutritious meals, and shelter individuals who otherwise may experience homelessness away from the elements and traumatic experiences associated with homelessness.^24,25,26^ Unaffordable housing may mean choosing between paying rent and other necessities, including seeking health care services and filling prescriptions.^24,25,26^

Associations between police expenditures and health have rarely been examined, and the findings were mixed in a 2005 study on associations between state-level expenditures on police and corrections (combined total) and all-cause mortality.^11^ We build on this research by studying municipal-level, rather than state-level, police expenditures in New Jersey, a state with one of the largest numbers of municipal police departments in the US. Modern models of policing embrace a preemptive approach that involves police officers stopping individuals thought to be engaging in suspicious activity.^27^ Therefore, exposure to police can be a stressful or even traumatic event that is associated with poor self-rated health, diabetes, hypertension, obesity, depression, anxiety, and symptoms of posttraumatic stress disorder, particularly for individuals who are Black, Indigenous, and people of color, even when encounters with police do not involve physical or verbal violence or when the police exposure is vicarious, as in the case of mothers with youth who experienced police stops.^27,28,29,30^ A 2017 study^31^ found that the anticipation of one’s children experiencing racial discrimination, eg, at the hands of police, created psychosocial stress that was associated with adverse birth outcomes among pregnant women.

Overall, this study, which focused on a populous and diverse state that has one of the highest rates of SMM in the US, contributes to the scant to nonexistent previous research on area-level determinants of SMM and linkages between municipal expenditures and health. The large number of municipalities and births and high rate and geographic variation in SMM in New Jersey provided a valuable context within which to explore the geographic patterning of SMM and estimate associations between salient categories of local expenditures and SMM.

### Limitations

This study has several limitations. One limitation of our analysis is that, while we control for county-level spending on corresponding categories as well as for municipal-level deprivation and other relevant municipal-level characteristics, this study is not designed to assess whether observed associations between types of municipal spending and SMM are causal. However, the findings provide robust and important first-order information about the associations and point to potentially fruitful areas for future research. Another limitation is that by considering municipal expenditures and not actual services provided, we are not able to study different types of programs within expenditure categories. Future research that identifies the effects on SMM of specific municipal programs, eg, public housing vs housing vouchers in the case of housing expenditures, is warranted. Furthermore, we assumed mothers resided in the same municipality throughout the prenatal period and earlier. We also did not capture antepartum SMM, information from outpatient or emergency department visits, or access to care. Additionally, our results are from a single state, New Jersey, and thus our findings may not be generalizable to all US states.

## Conclusions

The findings from this cross-sectional study indicate that place was associated with SMM outcomes. The associations between spending on various types of services at the municipal level and SMM, holding constant the overall level of municipal spending and municipal socioeconomic status, suggest that municipal budget allocation decisions can have consequences for SMM rates. A major objective of the Maternal Health Action Plan of the US Department of Health and Human Services, released in 2020, was to enhance the targeting of resources by identifying problem spots for maternal morbidity and mortality. Our findings strongly suggest that surveillance at the municipal level, a level rarely considered in studies of health outcomes, would be important for success in such efforts.

## Notes

Supplement. **eTable 1.** Severe Maternal Morbidity Indicators, Associated *ICD-9-CM* or *ICD-10-CM* Codes, and Rates in New Jersey From 2008 to 2018 **eAppendix 1.** Municipality Deprivation Measure **eAppendix 2.** Smoothing Rates in Choropleth Maps **eAppendix 3.** Testing for Spatial Autocorrelation **eTable 2.** US Census Bureau Definition of Expenditure Categories **eTable 3.** Odds Ratios From Multilevel Models Examining Associations Between Severe Maternal Morbidity and Per Capita Municipal Government Expenditures in New Jersey From 2008 to 2018 **eTable 4.** Odds Ratios From Multilevel Models Examining Associations Between Severe Maternal Morbidity (Excluding Blood Transfusion) and Individual- and Municipality-Level Characteristics in New Jersey From 2008 to 2018 **eTable 5.** Odds Ratios From Multilevel Models Examining Associations Between Severe Maternal Morbidity (Excluding Blood Transfusion) and Per Capita Municipal Government Expenditures in New Jersey From 2008 to 2018 **eTable 6.** Odds Ratios From Multilevel Models Examining Associations Between Blood Transfusion and Individual- and Municipality-Level Characteristics in New Jersey From 2008 to 2018 **eTable 7.** Odds Ratios From Multilevel Models Examining Associations Between Blood Transfusion and Per Capita Municipal Government Expenditures in New Jersey From 2008 to 2018 **eAppendix 4.** Alternative Model Specifications **eTable 8.** Sensitivity Analysis of Multilevel Models Examining Associations Between Severe Maternal Morbidity and Individual- and Municipality-Level Characteristics in New Jersey From 2008 to 2018 **eTable 9.** Odds Ratios From Multilevel Models Examining Associations Between Severe Maternal Morbidity and Recent Per Capita Municipal Government Expenditures in New Jersey From 2008 to 2018
eReferences
