# Supplementary material for: Municipality-Level Variation in Severe Maternal Morbidity and Association With Municipal Expenditures in New Jersey
Source: JAMA Netw Open. 2021 Nov 18;4(11):e2135161. doi: 10.1001/jamanetworkopen.2021.35161 (PMC8603080; doi:10.1001/jamanetworkopen.2021.35161)
Supplement: Supplement. — eTable 1. Severe Maternal Morbidity Indicators, Associated ICD-9-CM or ICD-10-CM Codes, and Rates in New Jersey From 2008 to 2018 eAppendix 1. Municipality Deprivation Measure eAppendix 2. Smoothing Rates in Choropleth Maps eAppendix 3. Testing for Spatial Autocorrelation eTable 2. US Census Bureau Definition of Expenditure Categories eTable 3. Odds Ratios From Multilevel Models Examining Associations Between Severe Maternal Morbidity and Per Capita Municipal Government Expenditures in New Jersey From 2008 to 2018 eTable 4. Odds Ratios From Multilevel Models Examining Associations Between Severe Maternal Morbidity (Excluding Blood Transfusion) and Individual- and Municipality-Level Characteristics in New Jersey From 2008 to 2018 eTable 5. Odds Ratios From Multilevel Models Examining Associations Between Severe Maternal Morbidity (Excluding Blood Transfusion) and Per Capita Municipal Government Expenditures in New Jersey From 2008 to 2018 eTable 6. Odds Ratios From Multilevel Models Examining Associations Between Blood Transfusion and Individual- and Municipality-Level Characteristics in New Jersey From 2008 to 2018 eTable 7. Odds Ratios From Multilevel Models Examining Associations Between Blood Transfusion and Per Capita Municipal Government Expenditures in New Jersey From 2008 to 2018 eAppendix 4. Alternative Model Specifications eTable 8. Sensitivity Analysis of Multilevel Models Examining Associations Between Severe Maternal Morbidity and Individual- and Municipality-Level Characteristics in New Jersey From 2008 to 2018 eTable 9. Odds Ratios From Multilevel Models Examining Associations Between Severe Maternal Morbidity and Recent Per Capita Municipal Government Expenditures in New Jersey From 2008 to 2018 eReferences [file jamanetwopen-e2135161-s001.pdf]

Supplemental Online Content

Muchomba FM, Teitler J, Kruse L, Reichman NE. Municipality-level variation in severe maternal morbidity and association with municipal expenditures in New Jersey. *JAMA Netw Open*. 2021;4(11):e2135161. doi:10.1001/jamanetworkopen.2021.35161

eTable 1. Severe Maternal Morbidity Indicators, Associated *ICD-9-CM* or *ICD-10-CM* Codes, and Rates in New Jersey From 2008 to 2018
eAppendix 1. Municipality Deprivation Measure
eAppendix 2. Smoothing Rates in Choropleth Maps
eAppendix 3. Testing for Spatial Autocorrelation
eTable 2. US Census Bureau Definition of Expenditure Categories
eTable 3. Odds Ratios From Multilevel Models Examining Associations Between Severe Maternal Morbidity and Per Capita Municipal Government Expenditures in New Jersey From 2008 to 2018
eTable 4. Odds Ratios From Multilevel Models Examining Associations Between Severe Maternal Morbidity (Excluding Blood Transfusion) and Individual- and Municipality-Level Characteristics in New Jersey From 2008 to 2018
eTable 5. Odds Ratios From Multilevel Models Examining Associations Between Severe Maternal Morbidity (Excluding Blood Transfusion) and Per Capita Municipal Government Expenditures in New Jersey From 2008 to 2018
eTable 6. Odds Ratios From Multilevel Models Examining Associations Between Blood Transfusion and Individual- and Municipality-Level Characteristics in New Jersey From 2008 to 2018
eTable 7. Odds Ratios From Multilevel Models Examining Associations Between Blood Transfusion and Per Capita Municipal Government Expenditures in New Jersey From 2008 to 2018
eAppendix 4. Alternative Model Specifications
eTable 8. Sensitivity Analysis of Multilevel Models Examining Associations Between Severe Maternal Morbidity and Individual- and Municipality-Level Characteristics in New Jersey From 2008 to 2018
eTable 9. Odds Ratios From Multilevel Models Examining Associations Between Severe Maternal Morbidity and Recent Per Capita Municipal Government Expenditures in New Jersey From 2008 to 2018
eReferences

This supplemental material has been provided by the authors to give readers additional information about their work.

© 2021 Muchomba FM et al. *JAMA Network Open.*

eTable 1. Severe Maternal Morbidity Indicators, Associated *ICD-9-CM* or *ICD-10-CM* Codes, and Rates in New Jersey From 2008 to 2018

Indicator	ICD-9	ICD-10	Rate
**Diagnostic Codes**			
1. Acute myocardial infarction	410.xx	I21.xx I22.x	1.1
2. Aneurysm	441.xx	I71.xx I79.0	0.4
3. Acute renal failure	584.5 584.6 584.7 584.8 584.9 669.3x	N17.x O90.4	11.7
4. Adult respiratory distress syndrome	518.5x 518.81 518.82 518.84 799.1	J80 J95.1 J95.2 J95.3 J95.82x J96.0x J96.2x R09.2	9.7
5. Amniotic fluid embolism	673.1x	O88.1x	0.4
6. Cardiac arrest/ventricular fibrillation	427.41 427.42 427.5	I46.x I49.0x	1.1
7. Disseminated intravascular coagulation	286.6 286.9 666.3x	D65 D68.8 D68.9 O72.3	25.5
8. Eclampsia	642.6x	O15. X	5.6
9. Heart failure/arrest during surgery or procedure	997.1	I97.12x I97.13x I97.710 I97.711	1.4
10. Puerperal cerebrovascular disorders	430.xx 431.xx 432.xx 433.xx 434.xx 436xx 437.xx 671.5x 674.0x 997.02	I60.xx- I68.xx O22.51 O22.52 O22.53 I97.81x I97.82x O87.3	6.5
11. Pulmonary edema or Acute heart failure	518.4 428.1 428.0 428.21 428.23 428.31 428.33 428.41 428.43	J81.0 I50.1 I50.20 I50.21 I50.23 I50.30 I50.31 I50.33 I50.40 I50.41 I50.43 I50.9	5.7
12. Severe anesthesia complications	668.0x 668.1x 668.2x	O74.0 O74.1 O74.2 O74.3 O89.0x O89.1 O89.2	1.5
13. Sepsis	038.xx 995.91 995.92 670.2x	O85 O86.04 T80.211A T81.4XXA T81.44xx R65.20 A40.x A41.x A32.7	10.7
14. Shock	669.1x 785.5x 995.0 995.4 998.0x	O75.1 R57.x R65.21 T78.2XXA T88.2 XXA T88.6 XXA T81.10XA T81.11XA T81.19XA	6.6
15. Sickle cell disease with crisis	282.42 282.62 282.64 282.69	D57.0x D57.21x D57.41x D57.81x	2.0
16. Air and thrombotic embolism	415.1x 673.0x 673.2x 673.3x 673.8x	I26.x O88.0x O88.2x O88.3x O88.8x	5.1
**Procedure Codes**			
17. Conversion of cardiac rhythm	99.6x	5A2204Z 5A12012	1.3
18. Blood products transfusion	99.0x	30230H0 30230K0 30230L0 30230M0 30230N0 30230P0 30230R0 30230T0 30230H1 30230K1 30230L1 30230M1 30230N1 30230P1 30230R1 30230T1 30233H0 30233K0	134.0

© 2021 Muchomba FM et al. *JAMA Network Open.*

		30233L0 30233M0 30233N0 30233P0 30233R0 30233T0 30233H1 30233K1 30233L1 30233M1 30233N1 30233P1 30233R1 30233T1 30240H0 30240K0 30240L0 30240M0 30240N0 30240P0 30240R0 30240T0 30240H1 30240K1 30240L1 30240M1 30240N1 30240P1 30240R1 30240T1 30243H0 30243K0 30243L0 30243M0 30243N0 30243P0 30243R0 30243T0 30243H1 30243K1 30243L1 30243M1 30243N1 30243P1 30243R1 30243T1 30250H0 30250K0 30250L0 30250M0 30250N0 30250P0 30250R0 30250T0 30250H1 30250K1 30250L1 30250M1 30250N1 30250P1 30250R1 30250T1 30253H0 30253K0 30253L0 30253M0 30253N0 30253P0 30253R0 30253T0 30253H1 30253K1 30253L1 30253M1 30253N1 30253P1 30253R1 30253T1 30260H0 30260K0 30260L0 30260M0 30260N0 30260P0 30260R0 30260T0 30260H1 30260K1 30260L1 30260M1 30260N1 30260P1 30260R1 30260T1 30263H0 30263K0 30263L0 30263M0 30263N0 30263P0 30263R0 30263T0 302633H1 30263K1 30263L1 30263M1 30263N1 30263P1 30263R1 30263T1	
19. Hysterectomy	68.3x-68.9x	0UT90ZZ 0UT94ZZ 0UT97ZZ 0UT98ZZ 0UT9FZZ	11.9
20. Temporary tracheostomy	31.1	0B110Z 0B110F 0B113 0B114	0.4
21. Ventilation	93.90 96.01 96.02 96.03 96.05	5A1935Z 5A1945Z 5A1955Z	3.9

Source: Codes are from the Centers for Disease Control and Prevention (CDC).[2] Rates are from authors' calculations using linked birth and hospital discharge records from the State of New Jersey.

Note: ICD = International Classification of Diseases. ICD-9 codes were used for years prior to 2016 and the ICD-10 codes for 2016 and later. Rates are per 10,000 births.

© 2021 Muchomba FM et al. *JAMA Network Open.*

eAppendix 1. Municipality Deprivation Measure

For a measure of municipal-level socioeconomic status, we used a deprivation index created and validated by Messer et al.[1] To create this measure, we used principal components analysis to combine the following eight municipal-level indicators of deprivation obtained from American Community Survey into a single measure, with the first principal component (which explained 62.5% of variability) retained: percent of males in management and professional occupations (loading on first principal component = -0.34), percent of housing units that are crowded (0.29), percent of households in poverty (0.39), percent of households on public assistance (0.35), percent of households earning <$30,000 per year (0.40), percent of families that are female-headed households with dependents (0.36), percent of people 25 years and older with less than a high school education (0.38), and the percent of labor force population that is unemployed (0.31). All eight variables had loadings greater than the 0.25 threshold commonly used[1] and were retained. Each municipality's factor score was standardized and used in the regression analysis.

© 2021 Muchomba FM et al. *JAMA Network Open.*

eAppendix 2. Smoothing Rates in Choropleth Maps

To avoid unstable estimates owing to relatively small numbers of births in some municipalities, we used empirical Bayes smoothing to generate choropleth maps.[2] In this approach, the estimated SMM prevalence in each municipality is affected by its own data, as well as by the data in the rest of the state. The extent to which the estimate is affected depends on how imprecise the estimated prevalence in the municipality is. Municipalities with small numbers of births are thus said to borrow strength from other municipalities while municipalities with large numbers of births are precise using their own data.

© 2021 Muchomba FM et al. *JAMA Network Open.*

eAppendix 3. Testing for Spatial Autocorrelation

We tested for the presence of spatial autocorrelation in the residuals of all our models by computing global Moran's I. We specified a queen contiguity spatial weights matrix, in which municipalities that shared a boundary or corner were considered neighbors. A Moran's I that is different from 0 indicates there is spatial dependence in the data that is unaccounted for in the analysis. None of the global Moran's I statistics calculated were statistically different from zero, indicating that spatial autocorrelation was not a concern.

© 2021 Muchomba FM et al. *JAMA Network Open.*

eTable 2. US Census Bureau Definition of Expenditure Categories

Expenditure category	Definition
Education	Operation, maintenance, and construction of public schools and facilities for elementary and secondary education, vocational-technical education, and other educational institutions.
Libraries	Establishment and provision of libraries for use by the general public and the technical and financial support of privately-operated libraries.
Public Welfare	Expenditures under public welfare programs for the benefit of individuals contingent upon their need or to private vendors for services and commodities on behalf of low-income or other means-tested beneficiaries.
Public Health	Provision of services for the conservation and improvement of public health, other than hospital care, and financial support of other governments' health programs.
Fire and Ambulance	Prevention, avoidance, and suppression of fires and provision of ambulance, medical, rescue, or auxiliary services provided by fire protection agencies.
Police	Expenditures for general police and other governmental departments that preserve law and order, protect persons and property from illegal acts, and work to prevent, control, investigate, and reduce crime.
Housing and Community Development	Construction, operation, and support of housing and redevelopment projects and other activities to promote or aid public and private housing and community development.
Parks and Recreation	Provision and support of recreational and cultural-scientific facilities maintained for the benefit of residents and visitors.
Transportation	Maintenance, operation, repair, and construction of highways, streets, roads, alleys, sidewalks, bridges, tunnels, ferry boats, viaducts, and related structures, and public parking facilities and public mass transit systems

Source: U.S. Bureau of the Census. Government Finance and Employment Classification Manual. 2006.

© 2021 Muchomba FM et al. *JAMA Network Open.*

eTable 3. Odds Ratios From Multilevel Models Examining Associations Between Severe Maternal Morbidity and Per Capita Municipal Government Expenditures in New Jersey From 2008 to 2018

Expenditure category:	Education	Libraries	Public Welfare	Public Health	Fire and ambulance	Police	Housing & Community development	Parks and Recreation	Transportation
**Fixed parameters**									
Race/ethnicity									
Non-Hispanic white	1.00	1.00	1.00	1.00	1.00	1.00	1.00	1.00	1.00
Non-Hispanic Black	1.96 (1.86 - 2.06)	1.97 (1.87 - 2.07)	1.95 (1.86 - 2.05)	1.96 (1.86 - 2.06)	1.96 (1.86 - 2.06)	1.96 (1.86 - 2.06)	1.95 (1.86 - 2.05)	1.96 (1.86 - 2.06)	1.97 (1.87 - 2.07)
Non-Hispanic Asian	1.12 (1.05 - 1.19)	1.13 (1.06 - 1.20)	1.12 (1.05 - 1.19)	1.12 (1.05 - 1.19)	1.12 (1.05 - 1.19)	1.12 (1.05 - 1.19)	1.12 (1.05 - 1.19)	1.12 (1.05 - 1.19)	1.13 (1.06 - 1.20)
Non-Hispanic other/mixed	1.31 (1.16 - 1.47)	1.31 (1.17 - 1.47)	1.31 (1.16 - 1.47)	1.31 (1.16 - 1.47)	1.31 (1.16 - 1.47)	1.31 (1.16 - 1.47)	1.31 (1.16 - 1.47)	1.30 (1.16 - 1.46)	1.32 (1.17 - 1.48)
Hispanic	1.20 (1.14 - 1.26)	1.20 (1.15 - 1.26)	1.20 (1.14 - 1.26)	1.20 (1.14 - 1.26)	1.20 (1.14 - 1.26)	1.20 (1.14 - 1.25)	1.20 (1.14 - 1.25)	1.20 (1.14 - 1.25)	1.21 (1.15 - 1.27)
Maternal age									
<20	0.98 (0.91 - 1.05)	0.98 (0.91 - 1.05)	0.98 (0.91 - 1.05)	0.98 (0.91 - 1.05)	0.98 (0.91 - 1.05)	0.98 (0.91 - 1.05)	0.98 (0.91 - 1.05)	0.98 (0.91 - 1.05)	0.98 (0.91 - 1.05)
20–34	1.00	1.00	1.00	1.00	1.00	1.00	1.00	1.00	1.00
≥35	1.39 (1.28 - 1.51)	1.39 (1.29 - 1.51)	1.39 (1.28 - 1.51)	1.39 (1.28 - 1.51)	1.39 (1.28 - 1.51)	1.39 (1.28 - 1.51)	1.39 (1.28 - 1.51)	1.39 (1.28 - 1.51)	1.39 (1.29 - 1.51)
Education									
Less than high school	1.00	1.00	1.00	1.00	1.00	1.00	1.00	1.00	1.00
High school	0.92 (0.87 - 0.96)	0.92 (0.87 - 0.96)	0.92 (0.87 - 0.96)	0.92 (0.87 - 0.96)	0.92 (0.87 - 0.96)	0.92 (0.87 - 0.96)	0.92 (0.87 - 0.96)	0.91 (0.87 - 0.96)	0.92 (0.87 - 0.96)
Some college	0.82 (0.78 - 0.87)	0.82 (0.78 - 0.87)	0.82 (0.78 - 0.87)	0.82 (0.78 - 0.87)	0.82 (0.78 - 0.87)	0.82 (0.78 - 0.87)	0.82 (0.78 - 0.87)	0.82 (0.78 - 0.87)	0.83 (0.78 - 0.87)
College or higher	0.75 (0.71 - 0.80)	0.75 (0.71 - 0.80)	0.75 (0.71 - 0.80)	0.75 (0.71 - 0.80)	0.75 (0.71 - 0.80)	0.75 (0.71 - 0.80)	0.75 (0.71 - 0.80)	0.75 (0.71 - 0.80)	0.76 (0.71 - 0.80)
Parity									
1	1.00	1.00	1.00	1.00	1.00	1.00	1.00	1.00	1.00
2	0.73 (0.70 - 0.76)	0.73 (0.70 - 0.76)	0.73 (0.70 - 0.76)	0.73 (0.70 - 0.76)	0.73 (0.70 - 0.76)	0.73 (0.70 - 0.76)	0.73 (0.70 - 0.76)	0.73 (0.70 - 0.76)	0.73 (0.70 - 0.76)
≥3	1.12	1.12	1.12	1.12	1.12	1.12	1.12	1.12	1.12

© 2021 Muchomba FM et al. *JAMA Network Open.*

	(1.08 - 1.17)	(1.08 - 1.17)	(1.08 - 1.17)	(1.08 - 1.17)	(1.08 - 1.17)	(1.08 - 1.17)	(1.08 - 1.17)	(1.08 - 1.17)	(1.08 - 1.16)
Municipality-level variables									
Expenditure in category per capita	0.96 (0.91 - 1.01)	0.33 (0.15 - 0.72)	0.31 (0.03 - 2.95)	0.26 (0.06 - 1.07)	0.65 (0.46 - 0.91)	1.15 (1.04 - 1.28)	0.64 (0.48 - 0.85)	1.08 (0.76 - 1.52)	0.64 (0.43 - 0.94)
Deprivation	1.09 (1.06 - 1.12)	1.08 (1.05 - 1.11)	1.08 (1.05 - 1.12)	1.08 (1.05 - 1.11)	1.10 (1.07 - 1.13)	1.10 (1.07 - 1.13)	1.12 (1.09 - 1.16)	1.08 (1.05 - 1.11)	1.09 (1.06 - 1.12)
Total expenditure per capita	1.00 (0.97 - 1.02)	0.98 (0.96 - 1.00)	0.98 (0.96 - 1.00)	0.99 (0.97 - 1.01)	1.00 (0.97 - 1.02)	0.96 (0.93 - 0.98)	0.98 (0.96 - 1.00)	0.98 (0.96 - 1.00)	0.99 (0.97 - 1.01)
**Random parameters**									
Mother-level variance	1.95 (1.77 - 2.15)	1.95 (1.77 - 2.15)	1.95 (1.77 - 2.15)	1.95 (1.77 - 2.15)	1.95 (1.77 - 2.15)	1.95 (1.77 - 2.15)	1.95 (1.77 - 2.15)	1.95 (1.77 - 2.15)	1.95 (1.77 - 2.15)
Municipality-level variance	0.03 (0.02 - 0.05)	0.03 (0.02 - 0.04)	0.03 (0.02 - 0.04)	0.03 (0.02 - 0.04)	0.03 (0.02 - 0.04)	0.03 (0.03 - 0.05)	0.03 (0.02 - 0.04)	0.03 (0.02 - 0.04)	0.03 (0.02 - 0.04)
Median odds ratio (municipality)	1.19	1.19	1.19	1.18	1.19	1.19	1.19	1.18	1.18

Note: Models also control for year (categories for each year between 2008-2018), municipality population size, and county expenditure in category. Expenditures are in constant 2019 thousands of dollars. Data for municipal expenditures are shaded for ease of interpretation.

© 2021 Muchomba FM et al. *JAMA Network Open.*

eTable 4. Odds Ratios From Multilevel Models Examining Associations Between Severe Maternal Morbidity (Excluding Blood Transfusion) and Individual- and Municipality-Level Characteristics in New Jersey From 2008 to 2018[a]

	OR, 95% CI									
	Model 1		Model 2		Model 3		Model 4		Model 5	
**Fixed parameters**										
Race/ethnicity										
Non-Hispanic white			1.00		1.00				1.00	
Non-Hispanic Black			2.00	1.87 - 2.14	1.98	1.85 - 2.12			1.95	1.81 - 2.09
Non-Hispanic Asian			0.92	0.84 - 1.00	0.96	0.88 - 1.05			0.96	0.88 - 1.05
Non-Hispanic other/mixed			1.33	1.13 - 1.57	1.33	1.13 - 1.58			1.32	1.12 - 1.56
Hispanic			1.06	1.00 - 1.13	1.06	0.99 - 1.13			1.05	0.98 - 1.12
Maternal age										
<20					1.15	1.02 - 1.29			1.15	1.02 - 1.30
20–34					1.00				1.00	
≥35					1.95	1.71 - 2.21			1.95	1.72 - 2.22
Education										
Less than high school					1.00				1.00	
High school					1.06	0.98 - 1.15			1.07	0.98 - 1.16
Some college					0.96	0.88 - 1.05			0.97	0.89 - 1.06
College or higher					0.90	0.82 - 0.98			0.91	0.83 - 1.00
Parity										
1					1.00				1.00	
2					0.79	0.74 - 0.83			0.79	0.74 - 0.83
≥3					1.11	1.05 - 1.18			1.11	1.05 - 1.18
Municipality-level variables										
Deprivation							1.07	1.04 - 1.11	1.02	0.99 - 1.05
Total expenditure per capita							1.01	0.99 - 1.04	1.00	0.98 - 1.03
Population size							1.00	1.00 - 1.00	1.00	1.00 - 1.00
**Random parameters**										
Mother-level variance	2.38	2.00-2.83	2.29	1.92-2.74	2.28	1.92-2.72	2.38	2.00-2.83	2.29	1.92-2.72
Municipality-level variance	0.04	0.03-0.06	0.02	0.01-0.03	0.02	0.01-0.03	0.03	0.02-0.05	0.02	0.01-0.03
Median odds ratio (municipality)	1.22		1.14		1.15		1.20		1.15	

[a] OR = odds ratio, CI = confidence interval. Models also control for year of delivery (categories for each year between 2008-2018).

© 2021 Muchomba FM et al. *JAMA Network Open.*

eTable 5. Odds Ratios From Multilevel Models Examining Associations Between Severe Maternal Morbidity (Excluding Blood Transfusion) and Per Capita Municipal Government Expenditures in New Jersey From 2008 to 2018

Expenditure category:	Education	Libraries	Public Welfare	Public Health	Fire and ambulance	Police	Housing & Community development	Parks and Recreation	Transportation
**Fixed parameters**									
Race/ethnicity									
Non-Hispanic white	1.00	1.00	1.00	1.00	1.00	1.00	1.00	1.00	1.00
Non-Hispanic Black	1.95	1.95	1.95	1.96	1.96	1.96	1.95	1.95	1.96
	(1.81 - 2.10)	(1.82 - 2.10)	(1.81 - 2.10)	(1.82 - 2.11)	(1.82 - 2.11)	(1.82 - 2.11)	(1.81 - 2.09)	(1.81 - 2.10)	(1.82 - 2.10)
Non-Hispanic Asian	0.96	0.96	0.96	0.96	0.96	0.96	0.96	0.96	0.97
	(0.88 - 1.05)	(0.88 - 1.05)	(0.88 - 1.05)	(0.88 - 1.04)	(0.88 - 1.05)	(0.88 - 1.05)	(0.88 - 1.05)	(0.88 - 1.05)	(0.88 - 1.05)
Non-Hispanic other/mixed	1.32	1.33	1.32	1.32	1.33	1.33	1.32	1.32	1.33
	(1.12 - 1.56)	(1.12 - 1.57)	(1.12 - 1.57)	(1.12 - 1.57)	(1.12 - 1.57)	(1.12 - 1.57)	(1.12 - 1.56)	(1.12 - 1.57)	(1.12 - 1.57)
Hispanic	1.05	1.05	1.05	1.05	1.05	1.05	1.05	1.05	1.05
	(0.98 - 1.12)	(0.98 - 1.13)	(0.98 - 1.12)	(0.98 - 1.12)	(0.98 - 1.13)	(0.98 - 1.12)	(0.98 - 1.12)	(0.98 - 1.12)	(0.98 - 1.13)
Maternal age									
<20	1.15	1.15	1.15	1.15	1.15	1.15	1.15	1.15	1.15
	(1.02 - 1.30)	(1.02 - 1.30)	(1.02 - 1.30)	(1.02 - 1.30)	(1.02 - 1.30)	(1.02 - 1.30)	(1.02 - 1.30)	(1.02 - 1.30)	(1.02 - 1.30)
20–34	1.00	1.00	1.00	1.00	1.00	1.00	1.00	1.00	1.00
≥35	1.96	1.96	1.95	1.96	1.96	1.96	1.95	1.95	1.96
	(1.72 - 2.22)	(1.72 - 2.22)	(1.72 - 2.22)	(1.72 - 2.23)	(1.72 - 2.22)	(1.72 - 2.22)	(1.72 - 2.22)	(1.72 - 2.22)	(1.72 - 2.22)
Education									
Less than high school	1.00	1.00	1.00	1.00	1.00	1.00	1.00	1.00	1.00
High school	1.07	1.07	1.07	1.07	1.07	1.07	1.07	1.07	1.07
	(0.98 - 1.16)	(0.99 - 1.16)	(0.98 - 1.16)	(0.99 - 1.16)	(0.99 - 1.16)	(0.98 - 1.16)	(0.98 - 1.16)	(0.98 - 1.16)	(0.98 - 1.16)
Some college	0.97	0.97	0.97	0.97	0.97	0.97	0.97	0.97	0.97
	(0.89 - 1.06)	(0.89 - 1.06)	(0.89 - 1.06)	(0.89 - 1.06)	(0.89 - 1.06)	(0.89 - 1.06)	(0.89 - 1.06)	(0.89 - 1.06)	(0.89 - 1.06)
College or higher	0.91	0.92	0.91	0.92	0.92	0.91	0.91	0.91	0.92
	(0.84 - 1.00)	(0.84 - 1.00)	(0.83 - 1.00)	(0.84 - 1.00)	(0.84 - 1.00)	(0.84 - 1.00)	(0.84 - 1.00)	(0.83 - 1.00)	(0.84 - 1.00)
Parity									
1	1.00	1.00	1.00	1.00	1.00	1.00	1.00	1.00	1.00
2	0.79	0.79	0.79	0.79	0.79	0.79	0.79	0.79	0.79
	(0.74 - 0.83)	(0.74 - 0.83)	(0.74 - 0.83)	(0.75 - 0.83)	(0.74 - 0.83)	(0.75 - 0.83)	(0.74 - 0.83)	(0.74 - 0.83)	(0.74 - 0.83)
≥3	1.11	1.11	1.11	1.11	1.11	1.11	1.11	1.11	1.11
	(1.05 - 1.18)	(1.05 - 1.18)	(1.05 - 1.18)	(1.05 - 1.18)	(1.05 - 1.18)	(1.05 - 1.18)	(1.05 - 1.18)	(1.05 - 1.18)	(1.05 - 1.18)

© 2021 Muchomba FM et al. *JAMA Network Open.*

Municipality-level variables									
Expenditure in category per capita	0.97	1.46	0.37	0.28	0.64	1.13	0.86	1.15	0.92
	(0.92 - 1.02)	(0.62 - 3.43)	(0.04 - 3.54)	(0.06 - 1.28)	(0.44 - 0.92)	(1.01 - 1.27)	(0.64 - 1.16)	(0.75 - 1.76)	(0.59 - 1.43)
Deprivation	1.02	1.03	1.02	1.01	1.03	1.03	1.03	1.02	1.02
	(0.99 - 1.05)	(0.99 - 1.06)	(0.99 - 1.06)	(0.98 - 1.05)	(1.00 - 1.07)	(1.00 - 1.06)	(0.99 - 1.07)	(0.99 - 1.05)	(0.99 - 1.05)
Total expenditure per capita	1.02	1.00	1.01	1.02	1.03	0.99	1.01	1.00	1.01
	(0.99 - 1.05)	(0.98 - 1.03)	(0.98 - 1.03)	(0.99 - 1.04)	(1.00 - 1.06)	(0.97 - 1.02)	(0.99 - 1.03)	(0.98 - 1.03)	(0.99 - 1.03)
**Random parameters**									
Mother-level variance	2.29	2.29	2.29	2.28	2.28	2.28	2.29	2.29	2.28
	(1.88-2.69)	(1.88-2.69)	(1.88-2.69)	(1.88-2.68)	(1.88-2.69)	(1.88-2.68)	(1.89-2.69)	(1.89-2.69)	(1.88-2.69)
Municipality-level variance	0.02	0.02	0.02	0.02	0.02	0.02	0.02	0.02	0.02
	(0.01-0.03)	(0.01-0.03)	(0.01-0.03)	(0.01-0.03)	(0.01-0.03)	(0.01-0.03)	(0.01-0.03)	(0.01-0.03)	(0.01-0.03)
Median odds ratio (municipality)	1.15	1.15	1.15	1.15	1.15	1.15	1.15	1.15	1.15

Note: Models also control for year (categories for each year between 2008-2018), municipality population size, and county expenditure in category. Expenditures are in constant 2019 thousands of dollars. Data for municipal expenditures are shaded for ease of interpretation.

© 2021 Muchomba FM et al. *JAMA Network Open.*

eTable 6. Odds Ratios From Multilevel Models Examining Associations Between Blood Transfusion and Individual- and Municipality-Level Characteristics in New Jersey From 2008 to 2018[a]

	OR, 95% CI									
	Model 1		Model 2		Model 3		Model 4		Model 5	
**Fixed parameters**										
Race/ethnicity										
Non-Hispanic white			1.00		1.00				1.00	
Non-Hispanic Black			2.31	2.18 - 2.46	2.15	2.02 - 2.29			2.08	1.95 - 2.21
Non-Hispanic Asian			1.23	1.15 - 1.33	1.31	1.22 - 1.41			1.30	1.20 - 1.40
Non-Hispanic other/mixed			1.49	1.30 - 1.72	1.42	1.24 - 1.63			1.39	1.21 - 1.60
Hispanic			1.54	1.46 - 1.62	1.37	1.30 - 1.45			1.33	1.26 - 1.41
Maternal age										
<20					0.93	0.85 - 1.01			0.93	0.85 - 1.01
20–34					1.00				1.00	
≥35					1.22	1.11 - 1.34			1.23	1.12 - 1.35
Education										
Less than high school					1.00				1.00	
High school					0.86	0.81 - 0.91			0.86	0.81 - 0.92
Some college					0.76	0.71 - 0.82			0.77	0.72 - 0.83
College or higher					0.68	0.63 - 0.73			0.70	0.65 - 0.75
Parity										
1					1.00				1.00	
2					0.71	0.67 - 0.74			0.71	0.68 - 0.74
≥3					1.20	1.15 - 1.26			1.20	1.15 - 1.26
Municipality-level variables										
Deprivation							1.27	1.22 - 1.31	1.13	1.09 - 1.18
Total expenditure per capita							0.97	0.94 - 1.00	0.96	0.94 - 0.99
Population size							1.00	1.00 - 1.00	1.00	1.00 - 1.00
**Random parameters**										
Mother-level variance	2.50	2.26-2.76	2.42	2.19-2.68	2.42	2.18-2.68	2.50	2.26-2.76	2.42	2.19-2.69
Municipality-level variance	0.14	0.11-0.17	0.09	0.07-0.12	0.08	0.06-0.11	0.07	0.05-0.09	0.07	0.05-0.09
Median odds ratio (municipality)	1.43		1.34		1.32		1.29		1.28	

[a] OR = odds ratio, CI = confidence interval. Models also control for year of delivery (categories for each year between 2008-2018).

© 2021 Muchomba FM et al. *JAMA Network Open.*

eTable 7. Odds Ratios From Multilevel Models Examining Associations Between Blood Transfusion and Per Capita Municipal Government Expenditures in New Jersey From 2008 to 2018

Expenditure category:	Education	Libraries	Public Welfare	Public Health	Fire and ambulance	Police	Housing & Community development	Parks and Recreation	Transportation
**Fixed parameters**									
Race/ethnicity									
Non-Hispanic white	1.00	1.00	1.00	1.00	1.00	1.00	1.00	1.00	1.00
Non-Hispanic Black	2.08 (1.95 - 2.21)	2.09 (1.96 - 2.23)	2.07 (1.95 - 2.21)	2.08 (1.96 - 2.22)	2.08 (1.95 - 2.21)	2.08 (1.95 - 2.21)	2.08 (1.95 - 2.21)	2.08 (1.95 - 2.21)	2.10 (1.97 - 2.23)
Non-Hispanic Asian	1.30 (1.21 - 1.40)	1.31 (1.22 - 1.42)	1.30 (1.21 - 1.40)	1.30 (1.20 - 1.40)	1.30 (1.20 - 1.40)	1.30 (1.20 - 1.40)	1.30 (1.20 - 1.40)	1.29 (1.20 - 1.39)	1.32 (1.22 - 1.42)
Non-Hispanic other/mixed	1.39 (1.21 - 1.60)	1.40 (1.22 - 1.61)	1.39 (1.21 - 1.60)	1.39 (1.21 - 1.60)	1.39 (1.21 - 1.60)	1.39 (1.21 - 1.60)	1.39 (1.21 - 1.60)	1.39 (1.21 - 1.60)	1.41 (1.22 - 1.62)
Hispanic	1.33 (1.26 - 1.41)	1.34 (1.27 - 1.42)	1.33 (1.26 - 1.41)	1.33 (1.26 - 1.41)	1.33 (1.26 - 1.41)	1.33 (1.26 - 1.41)	1.33 (1.26 - 1.41)	1.33 (1.26 - 1.41)	1.35 (1.27 - 1.43)
Maternal age									
<20	0.93 (0.85 - 1.01)	0.93 (0.85 - 1.01)	0.93 (0.85 - 1.01)	0.93 (0.85 - 1.01)	0.93 (0.85 - 1.01)	0.93 (0.85 - 1.01)	0.93 (0.85 - 1.01)	0.93 (0.85 - 1.01)	0.93 (0.85 - 1.01)
20–34	1.00	1.00	1.00	1.00	1.00	1.00	1.00	1.00	1.00
≥35	1.23 (1.12 - 1.35)	1.23 (1.12 - 1.35)	1.23 (1.12 - 1.35)	1.23 (1.12 - 1.35)	1.23 (1.12 - 1.35)	1.23 (1.12 - 1.35)	1.23 (1.12 - 1.35)	1.23 (1.12 - 1.35)	1.23 (1.12 - 1.36)
Education									
Less than high school	1.00	1.00	1.00	1.00	1.00	1.00	1.00	1.00	1.00
High school	0.86 (0.81 - 0.92)	0.86 (0.81 - 0.92)	0.87 (0.82 - 0.92)	0.87 (0.82 - 0.92)	0.86 (0.81 - 0.92)	0.86 (0.81 - 0.92)	0.86 (0.81 - 0.92)	0.86 (0.81 - 0.92)	0.87 (0.82 - 0.92)
Some college	0.77 (0.72 - 0.83)	0.77 (0.72 - 0.83)	0.77 (0.72 - 0.83)	0.77 (0.72 - 0.83)	0.77 (0.72 - 0.83)	0.77 (0.72 - 0.83)	0.77 (0.72 - 0.83)	0.77 (0.72 - 0.83)	0.78 (0.72 - 0.83)
College or higher	0.70 (0.65 - 0.75)	0.70 (0.66 - 0.75)	0.70 (0.65 - 0.75)	0.70 (0.65 - 0.75)	0.70 (0.65 - 0.75)	0.70 (0.65 - 0.75)	0.70 (0.65 - 0.75)	0.70 (0.65 - 0.75)	0.70 (0.66 - 0.76)
Parity									
1	1.00	1.00	1.00	1.00	1.00	1.00	1.00	1.00	1.00
2	0.71 (0.67 - 0.74)	0.71 (0.68 - 0.74)	0.71 (0.67 - 0.74)	0.71 (0.68 - 0.74)	0.71 (0.67 - 0.74)	0.71 (0.68 - 0.74)	0.71 (0.68 - 0.74)	0.71 (0.68 - 0.74)	0.71 (0.67 - 0.74)
≥3	1.20	1.20	1.20	1.20	1.20	1.20	1.20	1.20	1.20

© 2021 Muchomba FM et al. *JAMA Network Open.*

	(1.15 - 1.26)	(1.15 - 1.26)	(1.15 - 1.26)	(1.15 - 1.26)	(1.15 - 1.26)	(1.15 - 1.26)	(1.15 - 1.26)	(1.15 - 1.26)	(1.14 - 1.26)
Municipality-level variables									
Expenditure in category per capita	0.95 (0.88 - 1.01)	0.10 (0.03 - 0.30)	0.37 (0.02 - 8.09)	0.32 (0.04 - 2.24)	0.72 (0.46 - 1.15)	1.12 (0.96 - 1.30)	0.56 (0.38 - 0.81)	1.16 (0.74 - 1.82)	0.67 (0.40 - 1.10)
Deprivation	1.13 (1.09 - 1.17)	1.11 (1.07 - 1.15)	1.12 (1.08 - 1.16)	1.12 (1.08 - 1.17)	1.13 (1.09 - 1.18)	1.14 (1.10 - 1.18)	1.17 (1.12 - 1.22)	1.11 (1.07 - 1.15)	1.13 (1.09 - 1.17)
Total expenditure per capita	0.99 (0.95 - 1.03)	0.98 (0.95 - 1.00)	0.96 (0.94 - 0.99)	0.97 (0.94 - 1.00)	0.98 (0.94 - 1.01)	0.95 (0.92 - 0.98)	0.97 (0.95 - 1.00)	0.97 (0.94 - 0.99)	0.98 (0.95 - 1.00)
**Random parameters**									
Mother-level variance	2.43 (2.19-2.69)	2.42 (2.19-2.69)	2.42 (2.19-2.69)	2.42 (2.19-2.69)	2.42 (2.19-2.69)	2.42 (2.19-2.69)	2.42 (2.19-2.69)	2.42 (2.19-2.68)	2.43 (2.19-2.69)
Municipality-level variance	0.07 (0.05-0.09)	0.06 (0.05-0.08)	0.06 (0.05-0.09)	0.07 (0.05-0.09)	0.07 (0.05-0.09)	0.07 (0.05-0.09)	0.07 (0.05-0.09)	0.05 (0.04-0.07)	0.06 (0.05-0.08)
Median odds ratio (municipality)	1.28	1.27	1.27	1.28	1.28	1.28	1.28	1.25	1.27

Note: Models also control for year (categories for each year between 2008-2018), municipality population size, and county expenditure in category. Expenditures are in constant 2019 thousands of dollars. Data for municipal expenditures are shaded for ease of interpretation.

© 2021 Muchomba FM et al. *JAMA Network Open.*

eAppendix 4. Alternative Model Specifications

Using recent municipal expenditures from the 2007, 2012, and 2017 Census of Governments yielded similar associations between total municipal expenditure per capita and SMM, as well as estimates of municipality-level variance (Models 1 and 2 of eTable 8). Further adjustment for smoking during pregnancy, delivery method, and pre-existing medical conditions (pre-pregnancy hypertension and pre-pregnancy diabetes) that are associated with SMM, left coefficients on the municipal-level characteristics unchanged (Model 3 of eTable 8).

Associations between different categories of recent municipal expenditures and SMM (presented in eTable 9) were in the same direction as those estimated in our primary models, although the effect sizes were often smaller, which could be because regression models using recent measures of expenditures capture short-term associations with SMM.

© 2021 Muchomba FM et al. *JAMA Network Open.*

eTable 8. Sensitivity Analysis of Multilevel Models Examining Associations Between Severe Maternal Morbidity and Individual- and Municipality-Level Characteristics in New Jersey From 2008 to 2018[a]

	OR, 95% CI					
	Model 1		Model 2		Model 3	
**Fixed parameters**						
Race/ethnicity						
Non-Hispanic white			1.00		1.00	
Non-Hispanic Black			1.95	1.86 - 2.06	1.82	1.73 - 1.91
Non-Hispanic Asian			1.12	1.05 - 1.19	1.10	1.03 - 1.17
Non-Hispanic other/mixed			1.31	1.16 - 1.47	1.28	1.14 - 1.43
Hispanic			1.20	1.14 - 1.25	1.17	1.12 - 1.23
Maternal age						
<20			0.98	0.91 - 1.05	0.81	0.76 - 0.88
20–34			1.00		1.00	
≥35			1.39	1.28 - 1.50	0.97	0.90 - 1.06
Education						
Less than high school			1.00		1.00	
High school			0.92	0.87 - 0.96	0.88	0.84 - 0.93
Some college			0.82	0.78 - 0.87	0.78	0.74 - 0.83
College or higher			0.75	0.71 - 0.80	0.72	0.68 - 0.76
Parity						
1			1.00		1.00	
2			0.73	0.70 - 0.76	0.73	0.70 - 0.76
≥3			1.12	1.08 - 1.17	1.16	1.11 - 1.20
Delivery method						
Vaginal					1.00	
Forceps					3.08	2.53 - 3.75
Vacuum					1.69	1.51 - 1.89
Cesarean					3.37	3.26 - 3.49
Maternal smoking in pregnancy					1.05	0.99 - 1.12
Maternal pre-pregnancy diabetes					1.44	1.28 - 1.63
Maternal pre-pregnancy hypertension					1.77	1.62 - 1.93
Municipality-level variables						
Deprivation	1.20	1.16 - 1.23	1.09	1.06 - 1.13	1.10	1.07 - 1.12
Total expenditure per capita	0.99	0.97 - 1.01	0.98	0.96 - 1.00	0.97	0.96 - 0.99
Population size	1.00	1.00 - 1.00	1.00	1.00 - 1.00	1.00	1.00 - 1.00
**Random parameters**						
Mother-level variance	2.01	1.83 – 2.21	1.95	1.77 - 2.15	1.70	1.52 - 1.90
Municipality-level variance	0.04	0.03 – 0.05	0.03	0.03 - 0.05	0.03	0.02 - 0.04
Median odds ratio (municipality)	1.21		1.19		1.17	

[a] Municipal expenditures data are for 2007, 2012, and 2017. Individual-level data were matched to most recent expenditures data. OR = odds ratio, CI = confidence interval. Models also control for year of delivery (categories for each year between 2008-2018).

© 2021 Muchomba FM et al. *JAMA Network Open.*

eTable 9. Odds Ratios From Multilevel Models Examining Associations Between Severe Maternal Morbidity and Recent Per Capita Municipal Government Expenditures in New Jersey From 2008 to 2018

Expenditure category:	Education	Libraries	Public Welfare	Public Health	Fire and ambulance	Police	Housing & Community development	Parks and Recreation	Transportation
**Fixed parameters**									
Race/ethnicity									
Non-Hispanic white	1.00	1.00	1.00	1.00	1.00	1.00	1.00	1.00	1.00
Non-Hispanic Black	1.96 (1.86 - 2.06)	1.97 (1.87 - 2.07)	1.96 (1.86 - 2.06)	1.96 (1.86 - 2.06)	1.96 (1.86 - 2.06)	1.96 (1.86 - 2.06)	1.95 (1.86 - 2.06)	1.96 (1.86 - 2.06)	1.96 (1.86 - 2.06)
Non-Hispanic Asian	1.12 (1.05 - 1.19)	1.13 (1.06 - 1.20)	1.12 (1.05 - 1.19)	1.12 (1.05 - 1.19)	1.12 (1.05 - 1.19)	1.12 (1.05 - 1.19)	1.12 (1.05 - 1.19)	1.12 (1.05 - 1.19)	1.13 (1.06 - 1.20)
Non-Hispanic other/mixed	1.31 (1.16 - 1.47)	1.31 (1.17 - 1.47)	1.31 (1.16 - 1.47)	1.31 (1.16 - 1.47)	1.31 (1.17 - 1.47)	1.31 (1.16 - 1.47)	1.31 (1.16 - 1.47)	1.30 (1.16 - 1.46)	1.31 (1.17 - 1.47)
Hispanic	1.20 (1.14 - 1.26)	1.21 (1.15 - 1.26)	1.20 (1.14 - 1.26)	1.20 (1.14 - 1.26)	1.20 (1.14 - 1.26)	1.20 (1.14 - 1.25)	1.20 (1.14 - 1.25)	1.20 (1.14 - 1.25)	1.20 (1.15 - 1.26)
Maternal age									
<20	0.98 (0.91 - 1.05)	0.98 (0.91 - 1.05)	0.98 (0.91 - 1.05)	0.98 (0.91 - 1.05)	0.98 (0.91 - 1.05)	0.98 (0.91 - 1.05)	0.98 (0.91 - 1.05)	0.98 (0.91 - 1.05)	0.98 (0.91 - 1.05)
20–34	1.00	1.00	1.00	1.00	1.00	1.00	1.00	1.00	1.00
≥35	1.39 (1.28 - 1.51)	1.39 (1.29 - 1.51)	1.39 (1.28 - 1.51)	1.39 (1.29 - 1.51)	1.39 (1.28 - 1.51)	1.39 (1.28 - 1.51)	1.39 (1.28 - 1.51)	1.39 (1.28 - 1.51)	1.39 (1.29 - 1.51)
Education									
Less than high school	1.00	1.00	1.00	1.00	1.00	1.00	1.00	1.00	1.00
High school	0.92 (0.87 - 0.96)	0.92 (0.87 - 0.96)	0.92 (0.87 - 0.96)	0.92 (0.87 - 0.96)	0.92 (0.87 - 0.96)	0.92 (0.87 - 0.96)	0.92 (0.87 - 0.96)	0.91 (0.87 - 0.96)	0.92 (0.87 - 0.96)
Some college	0.82 (0.78 - 0.87)	0.83 (0.78 - 0.87)	0.82 (0.78 - 0.87)	0.83 (0.78 - 0.87)	0.82 (0.78 - 0.87)	0.82 (0.78 - 0.87)	0.82 (0.78 - 0.87)	0.82 (0.78 - 0.87)	0.83 (0.78 - 0.87)
College or higher	0.75 (0.71 - 0.80)	0.76 (0.71 - 0.80)	0.75 (0.71 - 0.80)	0.75 (0.71 - 0.80)	0.75 (0.71 - 0.80)	0.75 (0.71 - 0.80)	0.75 (0.71 - 0.80)	0.75 (0.71 - 0.80)	0.75 (0.71 - 0.80)
Parity									
1	1.00	1.00	1.00	1.00	1.00	1.00	1.00	1.00	1.00
2	0.73	0.73	0.73	0.73	0.73	0.73	0.73	0.73	0.73

© 2021 Muchomba FM et al. *JAMA Network Open.*

	(0.70 - 0.76)	(0.70 - 0.76)	(0.70 - 0.76)	(0.70 - 0.76)	(0.70 - 0.76)	(0.70 - 0.76)	(0.70 - 0.76)	(0.70 - 0.76)	(0.70 - 0.76)
≥3	1.12<br>(1.08 - 1.17)	1.12<br>(1.08 - 1.17)	1.12<br>(1.08 - 1.17)	1.12<br>(1.08 - 1.17)	1.12<br>(1.08 - 1.17)	1.12<br>(1.08 - 1.17)	1.12<br>(1.08 - 1.17)	1.12<br>(1.08 - 1.17)	1.12<br>(1.08 - 1.16)
Municipality-level variables									
Expenditure in category per capita	0.97<br>(0.93 - 1.01)	0.35<br>(0.19 - 0.62)	0.07<br>(0.01 - 0.82)	0.31<br>(0.10 - 0.93)	0.64<br>(0.49 - 0.83)	1.01<br>(0.86 - 1.18)	0.88<br>(0.77 - 1.00)	1.01<br>(0.78 - 1.33)	0.86<br>(0.72 - 1.02)
Deprivation	1.09<br>(1.06 - 1.12)	1.08<br>(1.05 - 1.11)	1.09<br>(1.06 - 1.12)	1.09<br>(1.06 - 1.12)	1.10<br>(1.07 - 1.14)	1.10<br>(1.07 - 1.13)	1.10<br>(1.07 - 1.13)	1.08<br>(1.05 - 1.11)	1.09<br>(1.06 - 1.12)
Total expenditure per capita	0.99<br>(0.97 - 1.01)	0.99<br>(0.97 - 1.01)	0.98<br>(0.97 - 1.00)	0.99<br>(0.97 - 1.01)	1.00<br>(0.98 - 1.02)	0.98<br>(0.96 - 1.01)	0.99<br>(0.97 - 1.01)	0.99<br>(0.97 - 1.00)	0.99<br>(0.97 - 1.01)
**Random parameters**									
Mother-level variance	1.95<br>1.77 - 2.15	1.95<br>1.77 - 2.15	1.95<br>1.77 - 2.15	1.95<br>1.77 - 2.15	1.95<br>1.77 - 2.15	1.95<br>1.77 - 2.15	1.95<br>1.77 - 2.15	1.95<br>1.77 - 2.15	1.95<br>1.77 - 2.15
Municipality-level variance	0.03<br>0.02 - 0.04	0.03<br>0.02 - 0.04	0.03<br>0.02 - 0.04	0.03<br>0.02 - 0.04	0.03<br>0.02 - 0.04	0.03<br>0.03 - 0.05	0.03<br>0.03 - 0.05	0.03<br>0.02 - 0.04	0.03<br>0.02 - 0.04
Median odds ratio (municipality)	1.19	1.19	1.19	1.19	1.19	1.19	1.19	1.18	1.18

Note: Models also control for year (categories for each year between 2008-2018), municipality population size, and county expenditure in category. Municipal expenditures data are for 2007, 2012, and 2017. Individual-level data were matched to most recent expenditures data. Expenditures are in constant 2019 thousands of dollars. Data for municipal expenditures are shaded for ease of interpretation.

© 2021 Muchomba FM et al. *JAMA Network Open.*

eReferences

1. Messer L, Laraia B, Kaufman J, Eyster J, Holzman C, Culhane J, et al. The Development of a Standardized Neighborhood Deprivation Index. *J Urban Health*. 2006;83(6):1041-62.

2. Clayton D, Kaldor J. Empirical bayes estimates of age-standardized relative risks for use in disease mapping. *Biometrics*. 1987;43(3):671-681.

© 2021 Muchomba FM et al. *JAMA Network Open.*
